# Decreased MicroRNA-150 Exacerbates Neuronal Apoptosis in the Diabetic Retina

**DOI:** 10.3390/biomedicines9091135

**Published:** 2021-09-01

**Authors:** Fei Yu, Michael L. Ko, Gladys Y.-P. Ko

**Affiliations:** 1 Department of Veterinary Integrative Biosciences, College of Veterinary Medicine and Biomedical Sciences, Texas A&M University, College Station, TX 77843, USA; yufei00@tamu.edu (F.Y.); Michael.ko@blinn.edu (M.L.K.); 2 Department of Biology, Division of Natural and Physical Sciences, Blinn College, Bryan, TX 77802, USA; 3 Texas A&M Institute for Neuroscience, Texas A&M University, College Station, TX 77843, USA

**Keywords:** diabetes, diabetic retinopathy, retina, photoreceptor, apoptosis, microRNA

## Abstract

Diabetic retinopathy (DR) is a chronic complication associated with diabetes and the number one cause of blindness in working adults in the US. More than 90% of diabetic patients have obesity-associated type 2 diabetes (T2D), and 60% of T2D patients will develop DR. Photoreceptors undergo apoptosis shortly after the onset of diabetes, which contributes to the retinal dysfunction and microvascular complications leading to vision impairment. However, how diabetic insults cause photoreceptor apoptosis remains unclear. In this study, obesity-associated T2D mice and cultured photoreceptors were used to investigate how decreased microRNA-150 (miR-150) and its downstream target were involved in photoreceptor apoptosis. In the T2D retina, miR-150 was decreased with its target ETS-domain transcription factor (ELK1) and phosphorylated ELK1 at threonine 417 (pELK1_T417_) upregulated. In cultured photoreceptors, treatments with palmitic acid (PA), to mimic a high-fat environment, decreased miR-150 but upregulated ELK1, pELK1_T417_, and the translocation of pELK1_T417_ from the cytoplasm to the cell nucleus. Deletion of miR-150 (miR-150^−/−^) exacerbates T2D- or PA-induced photoreceptor apoptosis. Blocking the expression of ELK1 with small interfering RNA (siRNA) for *Elk1* did not rescue PA-induced photoreceptor apoptosis. Translocation of pELK1_T417_ from cytoplasm-to-nucleus appears to be the key step of diabetic insult-elicited photoreceptor apoptosis.

## 1. Introduction

Diabetic retinopathy (DR) is a chronic complication associated with type 1 and type 2 diabetes (T1D, T2D). It impacts 4.2 million people in the US and 93 million people worldwide [1]. About 95% of diabetic patients have T2D, and 60% of T2D patients develop DR in their lifetime [2]. Patients with DR have impaired neural retinas and vascular integrity, which leads to vision loss and blindness [3]. Intraocular injections of anti-vascular endothelial growth factor (VEGF) agents are currently the most effective therapy for inhibiting the angiogenesis seen in DR [4], but less than 50% of patients have improved vision after 1–2 years of anti-VEGF injections [5]. In general, the current therapies for DR mainly target the ocular neovascularization at the later stages of DR when visual function is already damaged and difficult to restore [6]. Therefore, it is important to investigate the pathological process in the neural retina at the early stages of DR to develop new therapeutic strategies in the future.

In the diabetic retina, neural dysfunction and degeneration occur early in the development of DR. Patients with T1D have thinner neural layers in the retina and visual dysfunction before the diagnostics of DR [7]. Adolescents with T2D for an average of two years have reduced retinal thickness and dampened light responses [8]. The loss of retinal neurons starts from 10 weeks after streptozotocin (STZ)-induced T1D in mice [9], and neuronal apoptosis in the retina occurs in T2D (db/db) mice from 20 weeks of age [10]. Among retinal neurons, photoreceptors undergo apoptosis shortly after the onset of diabetes [11,12]. Apoptotic photoreceptors can be detected in STZ rats 4 weeks after the onset of diabetes [11]. In addition, the dysfunction of photoreceptors in STZ mice is associated with the reduced thickness of the outer nuclear layer (ONL) [13]. Furthermore, diabetic patients with retinitis pigmentosa (RP), a genetic disease with loss of photoreceptors, rarely develop DR, even though these patients develop other diabetes-related vascular diseases [14,15]. In a mouse model of RP, during the period when photoreceptors are undergoing apoptosis, the retinal vasculature is also degenerating. Once the photoreceptors are completely lost, the vascular degeneration stops [16]. Hence, photoreceptor apoptosis not only contributes to the neural dysfunction under diabetes but may also adversely impact diabetic microvascular complications [16]. However, how diabetic insults cause photoreceptor apoptosis remains unclear.

MicroRNAs (miRs) represent a set of regulators that impact cell proliferation and apoptosis, and diabetes-associated changes in miR levels have been linked to the development of DR [17]. Among them, downregulation of miR-150 is observed in diabetic patients with DR [18], as well as in the plasma and retina of diabetic animals [19]. Inhibition of miR-150 promotes apoptosis [20], while overexpression of miR-150 alleviates the apoptosis of cells under hypoxia [21], and local hypoxia also occurs in the diabetic retina [22,23]. ETS-domain transcription factor (*Elk1*) is a confirmed target gene of miR-150 that is linked to apoptosis since overexpression of ELK1 protein has been found to promote apoptosis in neurons [24]. Transfection of *Elk1* in the dendrites of primary neurons induces apoptosis [25], while inhibition of *Elk1* alleviates the apoptosis of neurons under oxygen–glucose deprivation [26]. In addition, phosphorylated ELK1 at threonine 417 (pELK1_T417_) is essential for ELK1-mediated neuronal apoptosis [27]. However, it is unclear whether miR-150 and *Elk1* mediate neural apoptosis in the diabetic retina.

In our obesity-associated T2D mouse model induced by a high-fat diet (HFD), the miR-150 knockout (miR-150^−/−^) mice fed with the HFD showed more severe retinal neural dysfunction compared with the wild-type (WT) mice fed with the HFD [19,28]. In this study, we compared WT and miR-150^−/−^ mice fed with the HFD (60% fat calories) and determined the role of miR-150 and *Elk1* in photoreceptor apoptosis induced by obesity-associated T2DR. We further used cultured 661W cells, a mouse photoreceptor cell line [29], to decipher the relationship between miR-150, *Elk1*, ELK1, and photoreceptor apoptosis. We delineated the critical cellular localization of pELK1_T417_ and photoreceptor apoptosis under palmitic acid (PA), which mimics a high-fat environment for cells. Our data suggest that the cytoplasm-to-nucleus translocation of pELK1_T417_ could be the key step for photoreceptor apoptosis in obesity-associated T2DR.

## 2. Materials and Methods

### 2.1. Animals

Four-week-old male C57BL/6J mice (wild type, WT) were purchased from the Jackson Laboratory (Bar Harbor, ME, USA). B6(C)-Mir150tm1Rsky/J (miR-150^−/−^) mice were originally purchased from the Jackson Laboratory, and a colony was bred and maintained at Texas A&M University (College Station, TX, USA). Only male mice were used in this study. All animal experiments were approved by the Institutional Animal Care and Use Committee of Texas A&M University and were performed in compliance with the Association for Research in Vision and Ophthalmology (ARVO, Rockville, MD, USA) Statement for the Use of Animals in Ophthalmic and Vision Research. Mice were housed under temperature- and humidity-controlled conditions with 12:12 h of light–dark cycles. All mice were given food and water *ad libitum*. At 5 weeks of age (body weight, 20 g), mice were fed a high-fat diet (HFD; 60% fat calories, 20% protein calories, and 20% carbohydrate calories; #D12492; Research Diets, New Brunswick, NJ, USA) or a control diet (standard laboratory chow; 10% fat calories, 20% protein calories, and 70% carbohydrate calories; #D12450J; Research Diets) for up to 24 weeks. Bodyweight and food intake were measured weekly. Non-fasting blood glucose levels and glucose tolerance were measured monthly by taking blood from the tail vein. Glucose levels were measured using a Clarity BG1000 blood glucose monitoring system (Clarity Diagnostics, Boca Raton, FL, USA).

### 2.2. Cell Culture

The 661W cells [29] were originally obtained from Dr. Al-Ubaidi (University of Houston, Houston, TX, USA) and cultured in Dulbecco’s modified Eagle medium (DMEM; #12-614Q, Lonza, Portsmouth, NH, USA) containing 10% fetal bovine serum (FBS; #S11550, R&D Systems, Minneapolis, MN, USA), 2 mM Glutamax (#35050-061, Gibco/ThermoFisher, Waltham, MA, USA), 100 μg/mL penicillin and 100 μg/mL streptomycin (#15140-148, Gibco/Thermo Fisher, Waltham, MA, USA), and 1 mM sodium pyruvate (#S8636, Sigma, St. Louis, MO, USA) at 37 °C and 5% CO_2_. The 661W cells were treated with 100 µM palmitic acid (PA, #P5585-10G, Sigma, St. Louis, MO, USA) dissolved in 10% bovine serum albumin (BSA; #A6003-25G, Sigma, St. Louis, MO, USA) or an equal volume of 10% BSA (vehicle control) for various times as indicated.

### 2.3. Lipofectamine Transfection

Cells were transfected using the Lipofectamine 3000 kit (#L3000015, Invitrogen/Thermo Fisher, Waltham, MA, USA) according to the manufacturer’s instructions. Briefly, the 661W cells were seeded at 30% confluency and allowed to grow for 24 h to reach 50% confluency. For Western blot and quantitative real-time RT-PCR (qPCR), the cells were seeded in 6-well plates and transfected with 30 pmol/well microRNA (miRNA)/small interfering RNA (siRNA). For terminal deoxynucleotidyl transferase dUTP nick end labeling (TUNEL) and immunofluorescent staining, the cells were seeded on 12 mm circular coverslips in 24-well plates and transfected with 10 pmol/well miRNA/siRNA. After the first exchange to normal culture medium, some cultures were immediately treated with PA or BSA for various hours. The following miRNAs/siRNAs were used in this study: miRNA negative control (#4464058, Thermo Fisher), miR-150 mimic (Assay MC10070, #4464066, Thermo Fisher), miR-150 inhibitor (Assay MH10070, #4464084, Thermo Fisher), siRNA negative control (#AM4613, Thermo Fisher), and *Elk1* siRNA (Assay 261017, #AM16708, Thermo Fisher).

### 2.4. Terminal Deoxynucleotidyl Transferase dUTP Nick End Labeling (TUNEL)

The TUNEL staining was conducted according to the manufacturer’s instructions (#11684795910, Roche, Indianapolis, IN, USA). Briefly, cells cultured on coverslips were fixed with 4% paraformaldehyde for 1 h and permeabilized with 0.1% Triton X-100 in 0.1% sodium citrate at 4 °C for 5 min. After washing with phosphate buffered saline (PBS), 50 µL of TUNEL reaction mixture (5 µL enzyme + 45 µL label solution) was added to each coverslip and incubated at 37 °C in a humid dark chamber for 1 h. The slides were then washed with PBS and mounted with ProLong Gold antifade mountant with 4′,6′-diamidino-2-phenylindole (DAPI; #P36935, Thermo Fisher). The number of TUNEL positive (TUNEL^+^) 661W cells was counted from 10–15 regions for each culture well and normalized to the total number of cells. After deparaffinization, the retinal sections mounted on glass slides were immersed in 0.1 M citrate buffer (pH 6.0) and microwaved at 750 W for 1 min. After blocking the slides with 5% BSA and washing with PBS, TUNEL staining was applied as described above. Images were obtained using a Zeiss Axiovert 200M microscope (Carl Zeiss AG, Oberkochen, Germany). The number of TUNEL positive (TUNEL^+^) photoreceptors in the outer nuclear layer (ONL) of the retina was counted from 5–10 regions for each section and normalized to the ONL area.

### 2.5. The 3-[4,5-Dimethylthiazol-2-yl]-2,5 Diphenyl Tetrazolium Bromide (MTT) Colorimetric Assay

The 661W cells were seeded onto 96-well plates at 5.0 × 10^3^/well and allowed to grow for 24 h. After various experimental treatments, the proliferation/viability of 661W cells was determined by the MTT assay following the manufacturer’s protocol (Chem-Impex, Wood Dale, IL, USA). In brief, cells were incubated with the MTT solution (0.5 mg/mL final concentration) for 3 h at 37 °C until a purple precipitate was visible. After washing with PBS, 100 µL/well dimethylsulfoxide (DMSO) was added, and cells were kept in darkness at room temperature for 2 h to break the plasma membrane. The absorbance at 570 nm and the reference absorbance at 690 nm were measured with a spectrophotometer.

### 2.6. Quantitative Real-Time RT-PCR (qPCR)

After the cells were collected, total ribonucleic acid (RNA) from each sample was prepared by using a commercially available purification kit (miRNeasy mini kit; #217004, Qiagen, Germantown, MD, USA). From each sample, 500 ng of total RNA was used to quantify miR-150 or messenger RNAs (mRNAs) by qPCR using a TaqMan MicroRNA Reverse Transcription Kit (#4366596, Thermo Fisher) and SYBR green supermix ROX (#95055-500, QuantaBio, Beverly, MA, USA) with the CFX Connect Real-Time PCR Detection System (Bio-Rad, Hercules, CA, USA). The primers (Bioneer, Oakland, CA, USA) of *Elk1* (Forward 5′-GCC GGG CCT TGC GGT ACT ACT ATG A-3′, Reverse 5′-GGG TAG GAC ACA AAC TTG TAG AC-3′) and *β-actin* (Forward 5′-CAA CGG CTC CGG CAT GTG C-3′, Reverse 5′-GTA CAT GGC TGG GGT GTT GAA GGT C-3′) were used in this study.

For each experiment, a standard curve was generated with known quantities of RNAs loaded in curved dilutions (i.e., 2×, 1×, 1/2, 1/4, 1/8, 1/16, and 1/32). The cycle values, corresponding to the log values of the standard curve quantities, were used to generate a linear regression formula. The amplification efficiency of the qPCR reactions (90–100%) was calculated using the standard curve. The quantification of sample RNA was calculated by the 2^(−ΔΔCt)^ method [30] using *β-actin* as the internal control.

### 2.7. Western Blot

Samples for Western blots were collected, prepared, and analyzed as described previously [31,32]. Briefly, 661W cells were harvested and lysed in a Tris lysis buffer (in mM): 50 Tris, 1 EGTA, 150 NaCl, 1% Triton X-100, 1% β-mercaptoethanol, 50 NaF, and 1 Na_3_VO_4_, pH 7.5. Samples were separated on 10% sodium dodecyl sulfatepolyacrylamide gels by electrophoresis and transferred to nitrocellulose membranes. The membranes were blocked in 3% BSA in tris buffered saline Tween 20 (TBST) at room temperature for 1 h and incubated in primary antibodies overnight at 4 °C. After washing with TBST, the membranes were incubated in anti-rabbit IgG horseradish peroxidase (HRP)-linked secondary antibody (1:1000, #7074S, Cell Signaling, Beverly, MA, USA) at room temperature for 1 h. The blots were visualized using Super Signal West Pico/Femto chemiluminescent substrate (#34078/#34096, ThemoFisher). Band intensities were quantified using Image J (National Institutes of Health; NIH, Bethesda, MA, USA). The primary antibodies used in this study were anti-ELK1 (1:500, #9182S, Cell Signaling) and anti-β-actin (1:2000, #8457L, Cell Signaling). The band intensities of ELK1 were normalized to those of β-actin.

### 2.8. Immunofluorescent Staining (Retina and Cultured Cells)

Mouse eyes were collected, fixed with 4% paraformaldehyde, and processed for paraffin-embedded sectioning after 24 weeks of the diet regimen. Paraffin sections (4 μm) of the mouse eyes from all four experimental groups were mounted on the same glass slide. The retina sections were deparaffinized by heating at 57 °C followed by washing with xylene and serial dilutions of ethanol. Sections were then permeabilized in sodium citrate buffer (10 mM sodium citrate, 0.05% Tween 20, pH 6.0) at 80 °C for 1 h. The 661W cells cultured on coverslips were fixed with 4% paraformaldehyde at room temperature for 1 h and permeabilized with 0.1% Triton X-100 in 0.1% sodium citrate at 4 °C for 10 min.

Eye sections or coverslips were then blocked with 10% goat serum in PBS for 2 h at room temperature and incubated with primary antibodies overnight at 4 °C. After washing with PBS, sections or coverslips were incubated with secondary antibodies for 2 h at room temperature and mounted with ProLong Gold antifade mounted with DAPI. Images were obtained using a Zeiss Axiovert 200M microscope (Carl Zeiss AG, Jena, Germany). All fluorescent images were taken under identical settings including light intensity, exposure time, and magnification [19,31,33].

The fluorescent intensity was measured in the inner and outer segments of photoreceptors (IS + OS) and in the outer nuclear layer (ONL) for mouse retina sections or in the nuclear and cytoplasmic areas of 661W cells using ImageJ (NIH, Bethesda, MA, USA). The DAPI stain was used to identify the nuclear regions of the cells. The intensity of pELK1 in the cytoplasm was measured at the processes of photoreceptors that were 10 µm from the nucleus. The intensity of pELK1 in the nucleus was measured within the DAPI-stained area. We analyzed 10–15 regions for each culture well. The intensities of ELK1/pELK1 signal in the IS + OS and ONL were measured from 5–10 regions for each retinal section.

The following primary antibodies were used: anti-ELK1 (1:50, #ab131465, abcam, Waltham, MA, USA) and anti-phospho-ELK1 (T417; 1:50, #ab194795, abcam). The following secondary antibodies were used: goat anti-rabbit IgG (Alexa Fluor 488; 1:50, #ab150077, abcam, Irving, TX, USA) and goat anti-rabbit IgG (Alexa Fluor 568, 1:50, # ab175471, abcam).

### 2.9. Statistical Analysis

All data are presented as mean ± standard error of the mean (SEM). Student’s *t*-test or one-way analysis of variance (ANOVA) followed by Tukey’s *post hoc* tests were used for statistical analyses among groups. Throughout, *p* < 0.05 was regarded as significant. Origin 9.0 (OriginLab, Northampton, MA, USA) was used for statistical analyses.

## 3. Results

### 3.1. MicroRNA-150 Knockout (miR-150^−/−^) Exacerbates Apoptosis in the Diabetic Retina

We previously showed that mice develop obesity-associated T2D after 3 months under the HFD [19,33] and that these mice further develop diabetic vascular leakage and microvascular degeneration after 6 months of HFD [19,34,35], thus making our HFD (with 60% fat calories) mouse model suitable to study obesity-associated T2DR. As retinal neurodegeneration occurs early in the development of DR [8,10], photoreceptors undergo apoptosis shortly after the onset of diabetes [11]. Inhibition of miR-150 is known to promote apoptosis of cells under hypoxia [20], a condition also occurs in the early diabetic retina [22]. Since miR-150 is expressed in the retina, especially in photoreceptors, and since it is decreased in early diabetic retina and blood circulation [19], we postulated that decreased miR-150 might exacerbate the apoptosis of photoreceptors in the diabetic retina.

**Figure 1:**
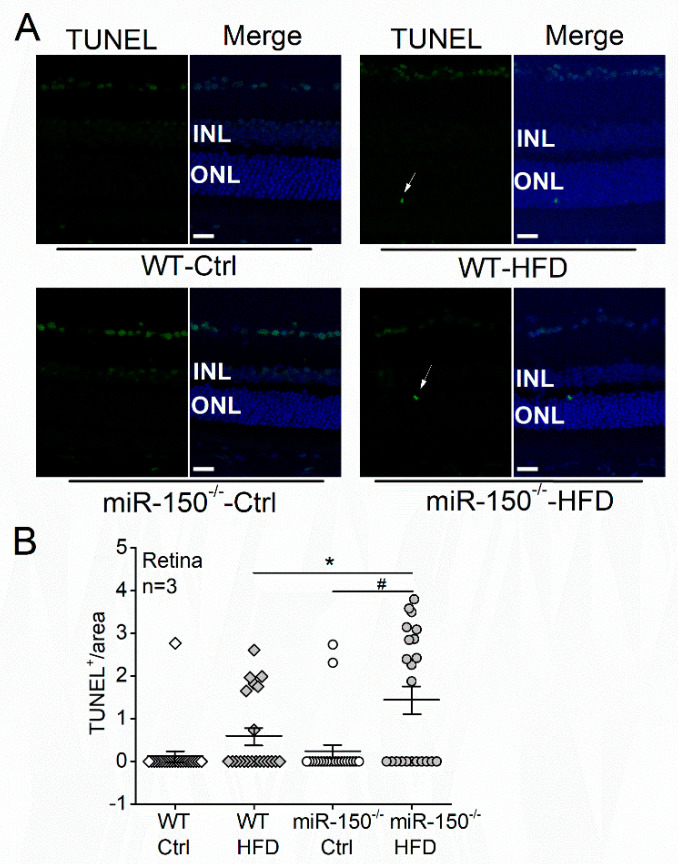
MicroRNA-150 knockout (miR-150^−/−^) exacerbates apoptosis in the diabetic retina. (**A**) Wild-type (WT) and miR-150^−/−^ mice were fed on normal chow (Ctrl) or high-fat diet (HFD) for 6 months. Terminal deoxynucleotidyl transferase dUTP nick end labeling (TUNEL) staining of mouse retinal sections shows TUNEL positive (TUNEL+) apoptotic cells in green fluorescence. The arrows indicate apoptotic photoreceptors in the outer nuclear layer (ONL). The 4′,6′-diamidino-2-phenylindole (DAPI; blue) stains the cell nuclei. Scale bar: 20 µm. (**B**) The number of TUNEL+ photoreceptors was counted from 5–10 regions for each retinal section and normalized to the ONL area. WT-Ctrl: open square; WT-HFD: gray square; miR-150^−/−^-Ctrl: open circle; miR-150^−/−^-HFD: gray circle. * and # indicate statistical significances specified with a horizontal line. Each group has *n* = 3 (mice). *p* < 0.05, one-way ANOVA.

Six months after the diet regimen, the wild-type (WT)-HFD mice had more TUNEL positive (TUNEL^+^) photoreceptors measured in the outer nuclear layer (ONL) than the WT mice with normal chow (WT-Ctrl; 0.59 ± 0.20 vs. 0.12 ± 0.12), even though there was no statistical significance (Figure 1). However, the miR-150^–/–^-HFD mouse retinas had significantly more TUNEL^+^ photoreceptors than the WT-HFD mice and mice under the normal diet (Ctrl; Figure 1). These results indicate that HFD induces more severe apoptosis of photoreceptors in the miR-150^−/−^ mice than in the WT mice, so photoreceptor apoptosis under T2D conditions was exacerbated by miR-150 knockout.

### 3.2. MicroRNA-150 Knockdown Exacerbates Palmitic Acid (PA)-Elicited Apoptosis in Cultured 661W Cells

**Figure 2:**
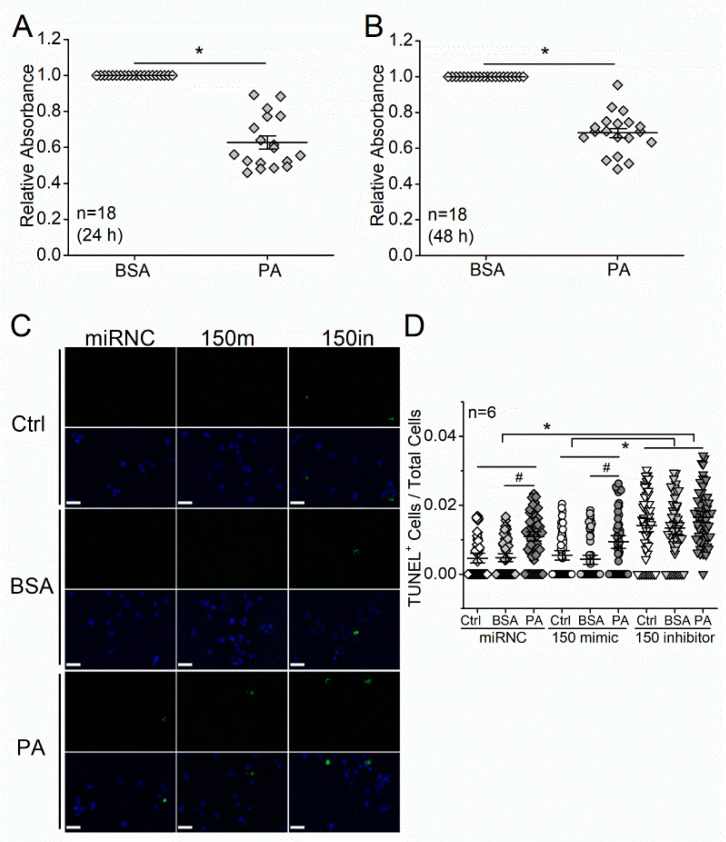
MicroRNA-150 knockdown exacerbates palmitic acid (PA)-elicited apoptosis in cultured 661W cells (a mouse photoreceptor cell line). The 661W cells were treated with 100 µM PA or BSA (vehicle control) for 24 h (**A**) or 48 h (**B**). and tested cell viability with the 3-[4,5-dimethylthiazol-2-yl]-2,5 diphenyl tetrazolium bromide (MTT) colorimetric assays. *n* = 18 for each group. *: *p* < 0.05, Student’s *t-*test. (**C**) The 661W cells were transfected with a microRNA negative control (miRNC), miR-150 mimic (150 m), or miR-150 inhibitor (150 in) and then treated with culture medium (Ctrl), BSA (vehicle control), or 100 µM PA (PA) for 24 h. Green fluorescence indicates TUNEL positive (TUNEL+), and the blue fluorescence is DAPI staining for cell nuclei. Scale bar: 30 µm. (**D**) The number of TUNEL+ 661W cells was counted from 10–15 regions for each culture well and normalized to the total number of cells. MiRNC-Ctrl: open diamond; miRNC-BSA: gray diamond; miRNC-PA: dark-diamond; 150 mimic-Ctrl: open circle; 150 mimic-BSA: gray circle; 150 mimic-PA: dark circle; 150 inhibitor-Ctrl: open triangle; 150 inhibitor-BSA: gray triangle; 150 inhibitor-PA: dark triangle. # indicates statistical difference to the BSA (vehicle). * indicates statistical difference between 150 inhibitor and miRNC, as well as 150 inhibitor and 150 mimic. *n* = 6 for each experimental group. *p* < 0.05, one-way ANOVA.

We next applied PA to cultured photoreceptors to mimic a high-fat environment in vitro and used MTT assays to examine the viability of cells. The 661W cells were originally derived from a mouse retinal tumor and characterized as a cone-photoreceptor cell line for expressing opsins, transducin, and arrestin [29]. The 661W cells treated with PA (100 µM) for 24 or 48 h showed decreased viability in MTT assays compared with cells treated with bovine serum albumin (BSA; vehicle control; Figure 2A,B). The decreased viability suggests increased apoptosis elicited by PA treatments.

As inhibition of miR-150 promotes cell apoptosis, we then tested whether knocking down miR-150 would exacerbate PA-elicited apoptosis in 661W cells. The 661W cells were first transfected with a miR negative control (miRNC), miR-150 mimic (150 m), or miR-150 inhibitor (150 in), then treated with culture medium (Ctrl), BSA (vehicle), or 100 µM PA for 24 h. Consistent with the earlier results (Figure 2A,B), cells treated with PA had increased apoptosis compared with the BSA- or Ctrl-treated cells transfected with 150 mimic and miRNC (Figure 2C,D). Interestingly, knocking down miR-150 in 661W cells (150 inhibitor) caused a significant increase in apoptosis regardless of the treatments (Figure 2D). However, transfection with miR-150 mimic did not attenuate the PA-induced apoptosis. These data suggest that overexpressing miR-150 in photoreceptors alone might not be enough to overturn the PA-induced apoptosis but that an adequate level of intrinsic miR-150 is necessary for the survival of photoreceptors.

### 3.3. ETS-Domain Transcription Factor 1 (Elk1) Is a Direct Target of miR-150 and Contributes to T2D-Induced Apoptosis in Photoreceptors

**Figure 3:**
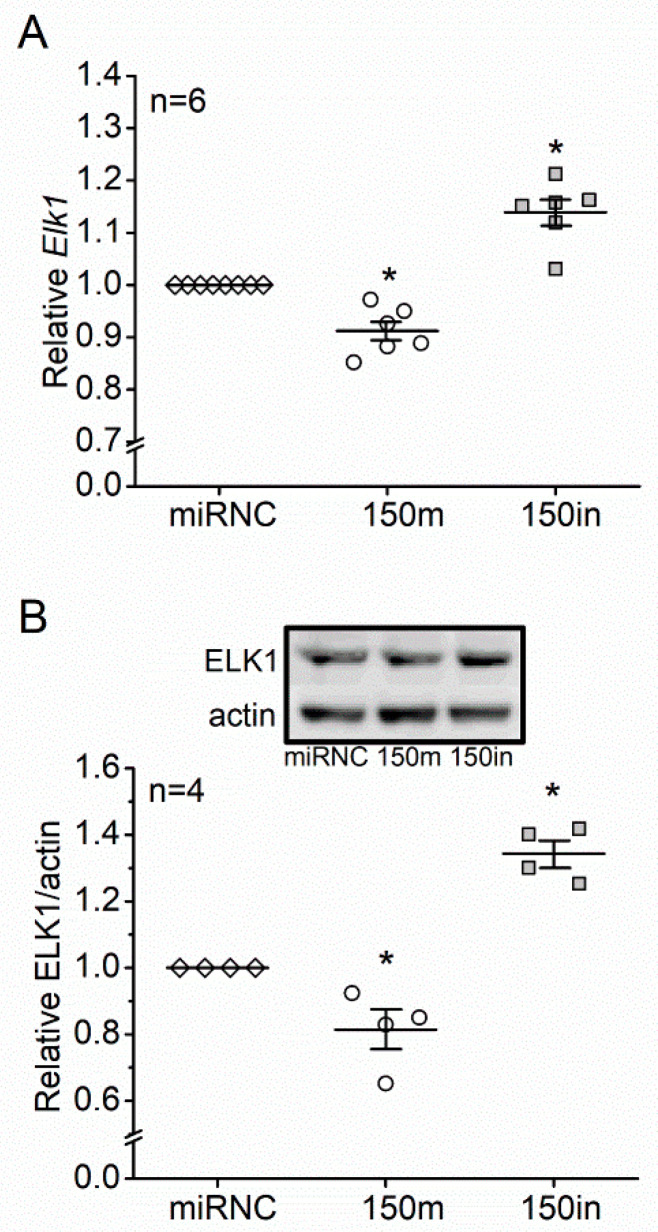
ETS-domain transcription factor 1 (*Elk1*) is a direct target of miR-150 in photoreceptors. The 661W cells were transfected with negative control (miRNC; open diamond), miR-150 mimic (150 m; open circle), or miR-150 inhibitor (150in; gray square) and collected to determine the levels of (**A**) *Elk1* mRNA using qPCR and (**B**) ELK1 protein using Western blots. * indicates statistical significance from the miRNC. *p* < 0.05, one-way ANOVA.

We then determined how miR-150 and its target genes regulate apoptosis in photoreceptors. MicroRNAs and their targets regulate bioactivities in a cell type- and tissue-specific manner [36,37]. Among the major targets of miR-150 expressed in photoreceptors, *Elk1* is known to promote apoptosis in neurons [24,25]. When 661W cells were transfected with a miR-150 mimic (150 m), the mRNA expression and protein level of *Elk1* were significantly decreased, while knocking down miR-150 (150 in) significantly increased the mRNA and protein of *Elk1* (Figure 3). We further examined the protein level of ELK1 and its phosphorylation at threonine 417 (pELK1_T417_) in the retinas from mice with six months of diet regimens since pELK1_T417_ (the active form of ELK1) is essential for the pro-apoptotic function of ELK1 [27].

**Figure 4:**
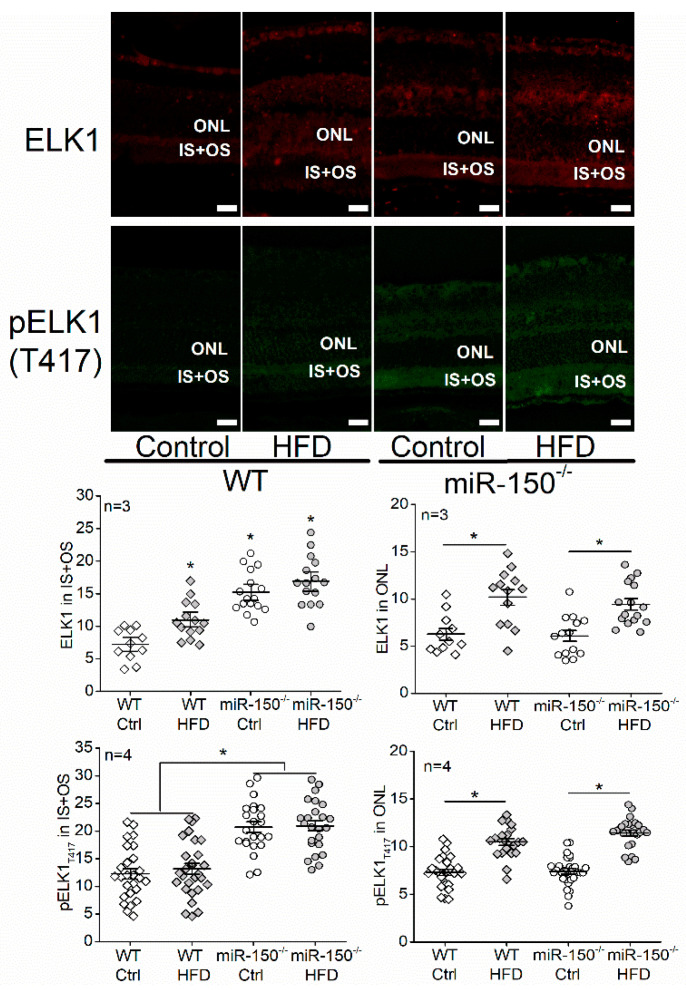
The high-fat diet (HFD) increases ELK1 (red) and phosphorylated ELK1 at T417 (pELK1_T417_; green) in the outer nuclear layer (ONL), while microRNA-150 knockout (miR-150^−/−^) upregulates ELK1 and pELK1_T417_ in the inner and outer segments of photoreceptors (IS + OS) after the WT and miR-150^−/−^ mice were fed a normal chow (Ctrl) or a high-fat diet (HFD) for 6 months. The signal intensities of ELK1 and pELK1_T417_ in the IS + OS and the ONL were measured. WT-Ctrl: open diamond; WT-HFD: gray diamond; miR-150^−/−^-Ctrl: open circle; miR-150^−/−^: gray circle. * *p* < 0.05, one-way ANOVA. Scale bar: 20 µm.

We specifically analyzed the levels of ELK1 and pELK1_T417_ in the inner and outer segments of photoreceptors (IS + OS) as well as in the outer nuclear layer (ONL). We found that the HFD regimen significantly increased the levels of ELK1 in the cytoplasm (IS + OS) and nuclei (ONL) of photoreceptors in WT and miR-150^−/−^ mice (Figure 4). Knockout of miR-150 (miR-150^−/−^) upregulated pELK1_T417_ in the IS + OS of photoreceptors, while HFD increased pELK1_T417_ in the ONL (Figure 4). It is possible that the HFD-induced apoptosis in photoreceptors (Figure 1) is mediated by an increase in nuclear pELK1_T417_ (Figure 4) and that the upregulated cytoplasmic pELK1_T417_ caused by miR-150 knockout (Figure 4) exacerbates the HFD-induced apoptosis (Figure 1).

### 3.4. Treatment with PA Increases ELK1 and Nuclear pELK1_T417_, and Knocking Down miR-150 Upregulates ELK1 and Cytoplasmic pELK1_T417_ in 661W Cells

**Figure 5:**
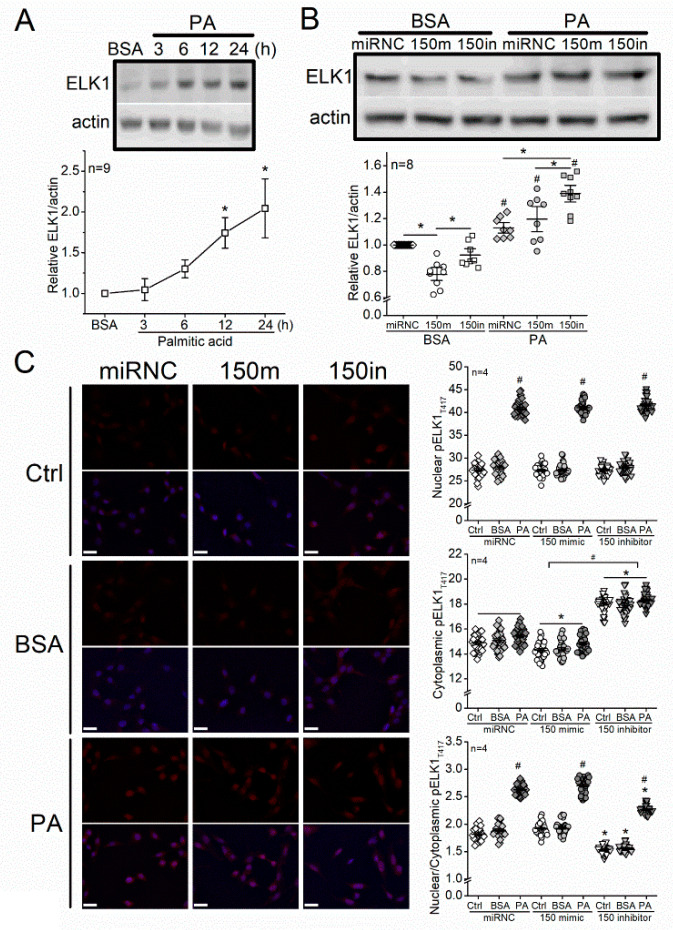
Treatment with PA increases ELK1 and nuclear pELK1_T417_, and knocking down miR-150 upregulates ELK1 and cytoplasmic pELK1_T417_ in 661W cells. (**A**) The 661W cells were treated with 100 µM PA or BSA (vehicle) for 3–24 h and then harvested to determine the protein levels of ELK1 via Western blots. * indicates a statistical significance from the BSA. *p* < 0.05, one-way ANOVA. (**B**) The 661W cells were first transfected with microRNA negative control (miRNC), miR-150 mimic (150 m), or miR-150 inhibitor (150 in) and then treated with BSA (vehicle) or 100 µM PA for 24 h. The ELK1 levels were determined by Western blots. miRNC-BSA: open diamond; 150 m-BSA: open circle; 150 in-BSA: open square; miRNC-PA: gray diamond; 150 m-PA: gray circle; 150 in-PA: gray square. * indicates a statistical significance when comparing among miRNC, 150m, and 150in. # indicates a statistical difference between PA and BSA. *p* < 0.05, one-way ANOVA. (**C**) The 661W cells were first transfected with microRNA negative control (miRNC), miR-150 mimic (150 m), or miR-150 inhibitor (150 in) and then treated with culture medium (Ctrl), BSA (vehicle), or 100 µM PA (PA) for 24 h. Cells were fixed and immunostained with pELK1_T417_ (red) and DAPI (blue). The fluorescent intensities of pELK1_T417_ in the nucleus or cytoplasm were quantified with ImageJ, and the intensity ratios of nuclear/cytoplasmic pELK1_T417_ were calculated. miRNC-Ctrl: open diamond; miRNC-BSA: gray diamond; miRNC-PA: dark diamond; 150 mimic-Ctrl: open circle; 150 mimic-BSA: gray circle; 150 mimic-PA: dark circle; 150 inhibitor-Ctrl: open triangle; 150 inhibitor-BSA: gray triangle; inhibitor-PA: dark triangle. For nuclear pELK1_T417_, # indicates a statistical significance when compared with BSA. For cytoplasmic pELK1_T417_, * indicates a statistical significance when compared with miRNC, and # indicates a statistical significance between 150 mimic and 150 inhibitor. For nuclear/cytoplasmic pELK1_T417_ ratio, # indicates a statistical significance when compared with BSA, and * indicates a statistical difference when compared with miRNC. *p* < 0.05, one-way ANOVA. Scale bar: 30 µm.

We further examined how ELK1 and pELK1_T417_ were altered by miR-150 and PA treatments in 661W cells. After cells were treated with PA, the levels of ELK1 were significantly increased in a time-dependent manner, compared with cells treated with BSA (vehicle; Figure 5A). The 661W cells were first transfected with microRNA negative control (miRNC), miR-150 mimic (150 m), or miR-150 inhibitor (150 in), and then treated with 100 µM PA for 24 h. As treatments with PA (24 h) significantly increased ELK1 in all cells, knocking down miR-150 (150 in) further elevated the PA-elicited increase in ELK1 (Figure 5B). Although transfection with miR-150 mimic (150 m) decreased ELK1 in cells treated with BSA (vehicle), it did not attenuate the PA-induced increase in ELK1 (Figure 5B), which suggests that overexpression of miR-150 is not sufficient to downregulate PA-stimulated ELK1.

Hence, the PA treatments elicited ELK1 upregulation (Figure 5A,B) and induced apoptosis (Figure 2) in 661W cells. Knocking down miR-150 further increased ELK1 (Figure 5B) and exacerbated the PA-induced apoptosis (Figure 2). However, transfection with miR-150 mimics did not attenuate the PA-induced increase in ELK1 (Figure 5B), and it did not overturn the PA-induced apoptosis (Figure 2). Using immunostaining, we found that cytoplasmic pELK1_T417_ was decreased in cells transfected with miR-150 mimic (150 m) but increased in cells transfected with miR-150 inhibitor (150 in) compared with the control (miRNC; Figure 5C). The PA-treated cells had increased nuclear pELK1_T417_ and nuclear/cytoplasmic (N/C) ratio of pELK1_T417_ compared with the BSA or control (Ctrl) group (Figure 5C).

### 3.5. Knocking Down Elk1 Decreases PA-Elicited Increases in Total ELK1 and pELK1_T417_ but Does Not Alleviate PA-Elicited Apoptosis in 661W Cells

**Figure 6:**
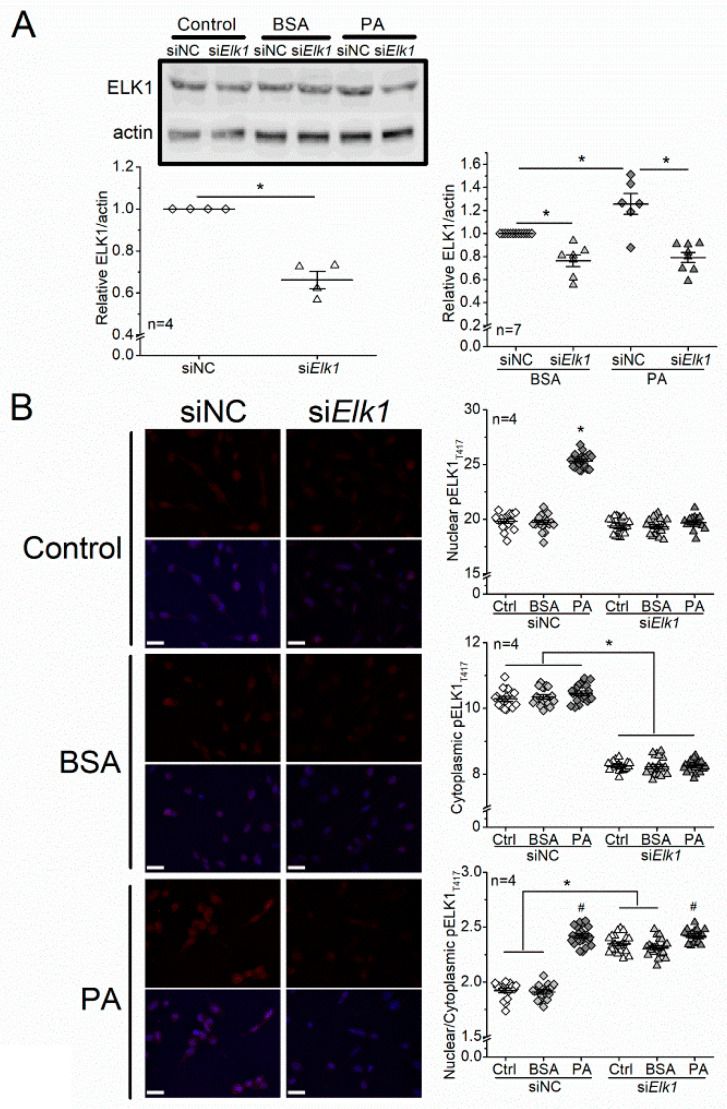
Knocking down *Elk1* decreases PA-elicited increases in total ELK1 and pELK1_T417_ in 661W cells. (**A**) The 661W cells were transfected with siRNA negative control (siNC) or *Elk1* siRNA (siElk1) and treated with culture medium (Ctrl), BSA, or 100 µM PA (PA) for 24 h. The ELK1 levels were determined by Western blots. siNC-Ctrl: open diamond; si*Elk1*-Ctrl: open triangle; siNC-BSA: gray diamond; siElk1-BSA: gray triangle; siNC-PA: dark diamond; siElk1-PA: dark triangle. The left panel * indicates a statistical significance between siNC and si*Elk1*; *p* < 0.05; Student’s *t*-test. The right panel * indicates a statistical significance between groups specified with a horizontal line; *p* < 0.05, one-way ANOVA. (**B**) The 661W cells were cultured on coverslips and transfected with siRNA negative control (siNC) or *Elk1* siRNA (si*Elk1*) and treated with culture medium (Ctrl), BSA, or 100 µM PA (PA) for 24 h. Cells were fixed and immunostained with pELK1_T417_ (red) and DAPI (blue). The fluorescent intensities of pELK1_T417_ in the nucleus or cytoplasm were quantified with ImageJ, and the intensity ratios of nuclear/cytoplasmic pELK1_T417_ were calculated. siNC-Ctrl: open diamond; siNC-BSA: gray diamond; siNC-PA: dark diamond; si*Elk1*-Ctrl: open triangle; si*Elk1*-BSA: gray triangle; si*Elk1*-PA: dark triangle. For nuclear pELK1_T417_, * indicates a statistical significance when compared with siNC-BSA. For cytoplasmic pELK1_T417_, * indicates a statistical significance between siNC and si*Elk1* regardless of treatments (Ctrl, BSA or PA). For nuclear/cytoplasmic pELK1_T417_ ratio, # indicates a statistical significance when compared with the BSA within siNC or si*Elk1*. * indicates a statistical significance between si*Elk1* and siNC specified with a horizontal line. *p* < 0.05, one-way ANOVA. Scale bar: 30 µm.

**Figure 7:**
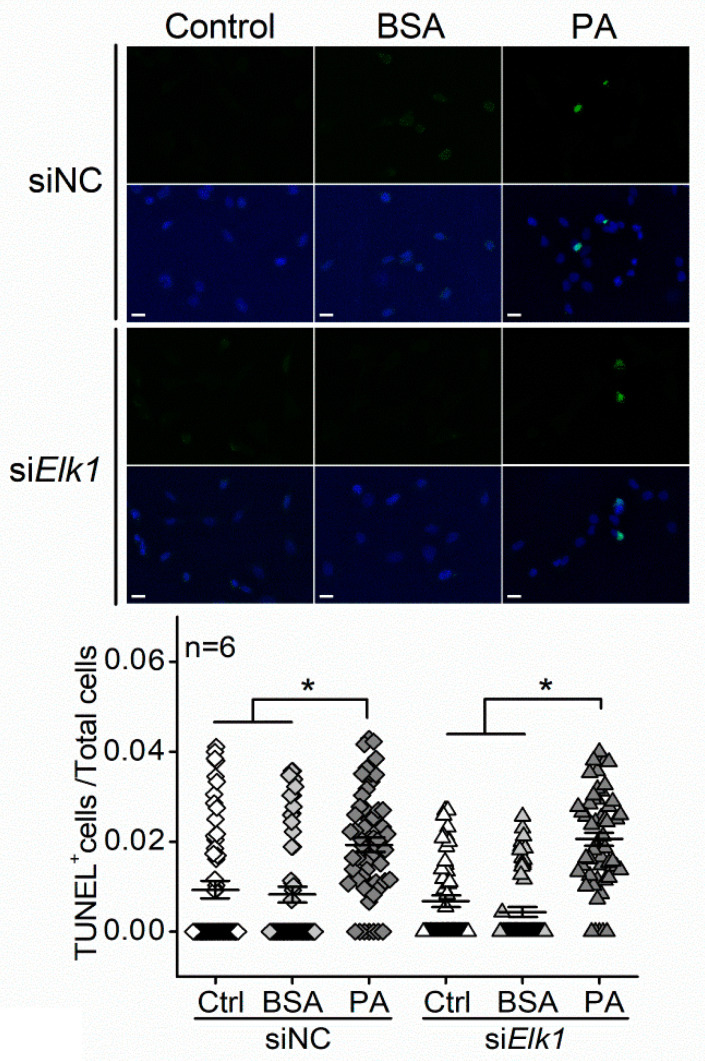
Knocking down *Elk1* does not alleviate PA-elicited apoptosis in 661W cells. The 661W cells were first transfected with siRNA negative control (siNC) or *Elk1* siRNA (si*Elk1*) and treated with culture medium (Ctrl), BSA, or 100 µM PA (PA) for 24 h. The TUNEL+ cells are green, and DAPI stained nuclei are blue. The ratio of TUNEL+ cells/total cells within each defined field was calculated. siNC-Ctrl: open diamond; siNC-BSA: gray diamond; siNC-BSA: dark diamond; si*Elk1*-Ctrl: open triangle; si*Elk1*-BSA: gray triangle; si*Elk1*-PA: dark triangle. * indicates a statistical significance. *n* = 6 experimental trials for each group. *p* < 0.05, one-way ANOVA. Scale bar: 30 µm.

In order to verify the functions of ELK1 and pELK1_T417_ in regulating apoptosis in photoreceptors, we knocked down *Elk1* in 661W cells by transfecting them with the small interfering RNA (siRNA) of *Elk1* (si*Elk1*). Cells transfected with si*Elk1* had decreased ELK1 expression compared with the cells transfected with a siRNA negative control (siNC). The PA-elicited increase in ELK1 was blocked by si*Elk1* (Figure 6A). In addition, knocking down *Elk1 (*si*Elk1)* decreased the cytoplasmic pELK1_T417_ and also arrested the PA-induced increase in nuclear pELK1_T417_ (Figure 6B). Thus, knocking down *Elk1* effectively inhibits the PA-elicited increase in ELK1 and pELK1_T417_. As we examined whether downregulation of *Elk1* could alleviate PA-induced apoptosis, we found that PA-induced increase in TUNEL^+^ cells was not dampened by transfection with si*Elk1* (Figure 7). The results indicate that knocking down *Elk1* did not attenuate PA-induced apoptosis.

## 4. Discussion

We previously found that miR-150 knockout exacerbated retinal neural dysfunction and vascular degeneration in obesity-associated T2DR in mice [19,28]. MiR-150 is decreased in the retina of DR patients and diabetic mice [19], but how this decrease contributes to the pathogenesis of DR is unknown. The neural retina has the highest oxygen consumption rate among all tissues, including the brain [38], thus making it (especially the retinal photoreceptors) prone to hypoxia-induced apoptosis. There is local hypoxia in the diabetic retina, and apoptosis of photoreceptors occurs shortly after the onset of diabetes and may contribute to neural dysfunction and vascular degeneration [13,16]. Inhibition of miR-150 is known to promote apoptosis [20,39], while overexpressing miR-150 can reduce the apoptosis of cells under hypoxia/ischemia conditions [21,40]. However, some cancer or endothelial cells with miR-150 overexpression have increased apoptosis under different treatments [41,42]. Thus, the function of miR-150 in regulating apoptosis might be tissue-specific and treatment-dependent, which might also be associated with its direct downstream targets. In this study, we report that the miR-150^−/−^-HFD mice had more severe apoptosis of photoreceptors than the WT-HFD mice (Figure 1), and knocking down miR-150 exacerbated the PA-induced apoptosis in 661W cells (Figure 2C,D). These results imply that decreased miR-150 exacerbates the apoptosis of photoreceptors under T2D conditions. Interestingly, knocking down miR-150 in 661 W cells caused a significant increase in apoptosis regardless of treatments (Figure 2D). This is partly different from the TUNEL staining results in the mouse retina, where the deletion of miR-150 (miR-150^−/−^) did not increase apoptosis without the HFD regimen (Figure 1). Since other cell types (e.g., Mϋller glial cells) in the retina may play a role in neuroprotection under stress, the retinal photoreceptors could be more resilient to the miR-150^−/−^ condition in vivo compared with isolated photoreceptors in culture. Alternatively, there might be some compensatory mechanisms against photoreceptor apoptosis during the development of T2DR in miR-150^−/−^ mice. This might require further investigations using conditional knockout animal models.

*Elk1* is a *bona fide* target of miR-150 [43]. We found that *Elk1* (mRNA) and ELK1 (protein) were decreased with miR-150 overexpression and increased with miR-150 knockdown in 661W cells (Figure 3A,B). The ELK1 expression was increased by T2D conditions (HFD or PA) in the mouse retina and 661W cells and was further increased by knocking down miR-150 (Figure 4 and Figure 5A,B). Overexpression of ELK1 is known to promote apoptosis in neurons [24,25], but apoptosis in most cancer cells is reduced by ELK1 overexpression [44,45,46]. As a transcription factor, ELK1 might regulate different downstream molecules in different types of cells/tissues and elicit paradoxical responses. We found that increased ELK1 (Figure 4 and Figure 5B) positively correlates with the increased apoptosis in photoreceptors of HFD mice (Figure 1) and in PA-treated 661W cells (Figure 2D), similar to neurons [24,25]. Knocking down *Elk1* was able to reduce the expression of ELK1 in PA-treated 661W cells (Figure 6A) but did not alleviate the PA-induced apoptosis (Figure 7). Thus, it is possible that the function of ELK1 in regulating apoptosis could be cell-type dependent. In retinal photoreceptors, downregulation of ELK1 might not be sufficient to completely overcome T2D-elicited apoptosis.

Phosphorylated/activated ELK1 has a positive correlation with increased apoptosis in neurons, but it is phosphorylation site-dependent [27]. Overexpression of WT *Elk1* or mutated *Elk1* (S383A) induced apoptosis in cultured primary neurons, but overexpression with a different mutated *Elk1* (T417A) does not induce apoptosis, which indicates that pELK1_T417_ is the active form of ELK1 that mediates neuronal apoptosis [27]. We found that the HFD regimen or PA treatments increased nuclear pELK1_T417_ and that miR-150 knockout or knockdown increased cytoplasmic pELK1_T417_ in mouse retinal photoreceptors or cultured 661W cells, respectively (Figure 4 and Figure 5C). There is a positive correlation between the HFD- or PA-elicited increase in nuclear pELK1_T417_ and increased photoreceptor apoptosis (Figure 1B and Figure 2D), which suggests that the T2D-induced increase in nuclear pELK1_T417_ could contribute to retinal photoreceptor apoptosis. Downregulation of miR-150 not only increases cytoplasmic pELK1_T417_ but also promotes apoptosis.

Apoptosis can be mediated by the mitochondrial permeability transition pore complex (PTP) that initiates mitochondrial swelling and membrane potential depolarization that leads to cell death [47]. There is a protein–protein interaction between ELK1 and PTP in the brain, and cytoplasmic ELK1 can be isolated from purified mitochondrial fractions. Furthermore, cell apoptosis induced by *Elk1* overexpression can be blocked by a PTP inhibitor in cultured primary neurons [24]. Thus, under T2D conditions, upregulated cytoplasmic pELK1_T417_ would have increased interactions with mitochondrial PTP, which might further accelerate photoreceptor apoptosis. However, while transfection with miR-150 mimics decreased cytoplasmic pELK1_T417_, it did not reduce nuclear pELK1_T417_ or overcome PA-elicited apoptosis. We noticed that the nuclear/cytoplasmic (N/C) ratio of pELK1_T417_ remained comparable between the 150m-PA and the miRNC-PA cells (Figure 5B) and between the si*Elk1*-PA and siNC-PA cells (Figure 6B). The N/C ratio represents the cytoplasm-to-nucleus translocation of pELK1_T417_, which is important for transactivating the downstream targets of *Elk1* and regulating apoptosis [48,49], as the translocation of ELK1 to the cell nucleus correlates with increased apoptosis in neurons [48]. Fos proto-oncogene (*c-Fos*) and myeloid cell leukemia 1 (*Mcl1*) are downstream targets of ELK1 that also mediate apoptosis [50,51]. Upregulated *c-Fos* promotes pathology-induced apoptosis in neurons, while downregulation of *c-Fos/Mcl1* alleviates apoptosis [50,51]. Therefore, blocking the translocation of pELK1_T417_ to the nucleus may be necessary to mitigate diabetes-associated apoptosis in photoreceptors.

## Data Availability

The data presented in this study are available from Fei Yu (F.Y.) and Gladys Y.-P. Ko (G.K.) upon reasonable request.

## References

[1] Barsegian A., Kotlyar B., Lee J., Salifu M.O., McFarlane S.I. (2017). Diabetic retinopathy: Focus on minority populations. Int. J. Clin. Endocrinol. Metab..

[2] Wong T.Y., Cheung C.M., Larsen M., Sharma S., Simo R. (2016). Diabetic retinopathy. Nat. Rev. Dis. Primers.

[3] Moran E.P., Wang Z., Chen J., Sapieha P., Smith L.E., Ma J.X. (2016). Neurovascular cross talk in diabetic retinopathy: Pathophysiological roles and therapeutic implications. Am. J. Physiol. Heart Circ. Physiol..

[4] Stewart M.W. (2016). Treatment of diabetic retinopathy: Recent advances and unresolved challenges. World J. Diabetes.

[5] Cheung N., Wong I.Y., Wong T.Y. (2014). Ocular anti-vegf therapy for diabetic retinopathy: Overview of clinical efficacy and evolving applications. Diabetes Care.

[6] Cheung N., Mitchell P., Wong T.Y. (2010). Diabetic retinopathy. Lancet.

[7] van Dijk H.W., Verbraak F.D., Stehouwer M., Kok P.H., Garvin M.K., Sonka M., DeVries J.H., Schlingemann R.O., Abramoff M.D. (2011). Association of visual function and ganglion cell layer thickness in patients with diabetes mellitus type 1 and no or minimal diabetic retinopathy. Vis. Res..

[8] Bronson-Castain K.W., Bearse M.A., Neuville J., Jonasdottir S., King-Hooper B., Barez S., Schneck M.E., Adams A.J. (2009). Adolescents with type 2 diabetes: Early indications of focal retinal neuropathy, retinal thinning, and venular dilation. RETINA.

[9] Martin P.M., Roon P., Van Ells T.K., Ganapathy V., Smith S.B. (2004). Death of retinal neurons in streptozotocin-induced diabetic mice. Invest Ophthalmol. Vis. Sci..

[10] Yang Q., Xu Y., Xie P., Cheng H., Song Q., Su T., Yuan S., Liu Q. (2015). Retinal neurodegeneration in db/db mice at the early period of diabetes. J. Ophthalmol..

[11] Park S.H., Park J.W., Park S.J., Kim K.Y., Chung J.W., Chun M.H., Oh S.J. (2003). Apoptotic death of photoreceptors in the streptozotocin-induced diabetic rat retina. Diabetologia.

[12] Zhang J., Wu Y., Jin Y., Ji F., Sinclair S.H., Luo Y., Xu G., Lu L., Dai W., Yanoff M. (2008). Intravitreal injection of erythropoietin protects both retinal vascular and neuronal cells in early diabetes. Invest Ophthalmol. Vis. Sci..

[13] Lv J., Bao S., Liu T., Wei L., Wang D., Ye W., Wang N., Song S., Li J., Chudhary M. (2020). Sulforaphane delays diabetes-induced retinal photoreceptor cell degeneration. Cell Tissue Res..

[14] Arden G.B. (2001). The absence of diabetic retinopathy in patients with retinitis pigmentosa: Implications for pathophysiology and possible treatment. Br. J. Ophthalm..

[15] Lahdenranta J., Pasqualini R., Schlingemann R.O., Hagedorn M., Stallcup W.B., Bucana C.D., Sidman R.L., Arap W. (2001). An anti-angiogenic state in mice and humans with retinal photoreceptor cell degeneration. Proc. Natl. Acad. Sci. USA.

[16] Liu H., Tang J., Du Y., Saadane A., Tonade D., Samuels I., Veenstra A., Palczewski K., Kern T.S. (2016). Photoreceptor cells influence retinal vascular degeneration in mouse models of retinal degeneration and diabetes. Invest Ophthalmol. Vis. Sci..

[17] Assmann T.S., Recamonde-Mendoza M., De Souza B.M., Crispim D. (2017). Microrna expression profiles and type 1 diabetes mellitus: Systematic review and bioinformatic analysis. Endocr. Connect..

[18] Mazzeo A., Beltramo E., Lopatina T., Gai C., Trento M., Porta M. (2018). Molecular and functional characterization of circulating extracellular vesicles from diabetic patients with and without retinopathy and healthy subjects. Exp. Eye Res..

[19] Yu F., Chapman S., Pham D.L., Ko M.L., Zhou B., Ko G.Y. (2020). Decreased mir-150 in obesity-associated type 2 diabetic mice increases intraocular inflammation and exacerbates retinal dysfunction. BMJ Open Diabetes Res. Care.

[20] Zhu J., Yao K., Guo J., Shi H., Ma L., Wang Q., Liu H., Gao W., Sun A., Zou Y. (2017). Mir-181a and mir-150 regulate dendritic cell immune inflammatory responses and cardiomyocyte apoptosis via targeting jak1-stat1/c-fos pathway. J. Cell Mol. Med..

[21] Ma J.L., Guo W.L., Chen X.M. (2018). Overexpressing microrna-150 attenuates hypoxia-induced human cardiomyocyte cell apoptosis by targeting glucose-regulated protein-94. Mol. Med. Rep..

[22] Wright W.S., McElhatten R.M., Messina J.E., Harris N.R. (2010). Hypoxia and the expression of hif-1alpha and hif-2alpha in the retina of streptozotocin-injected mice and rats. Exp. Eye Res..

[23] Linsenmeier R.A., Zhang H.F. (2017). Retinal oxygen: From animals to humans. Prog. Retin. Eye Res..

[24] Barrett L.E., Bockstaele E.J.V., Sul J.Y., Takano H., Haydon P.G., Eberwine J.H. (2006). Elk-1 associates with the mitochondrial permeablity transition pore complex in neurons. Proc. Natl. Acad. Sci. USA.

[25] Barrett L.E., Sul J.Y., Takano H., Van Bockstaele E.J., Haydon P.G., Eberwine J.H. (2006). Region-directed phototransfection reveals the functional significance of a dendritically synthesized transcription factor. Nat. Methods.

[26] Lu Z., Miao Z., Zhu J., Zhu G. (2018). Ets-domain containing protein (elk1) suppression protects cortical neurons against oxygen-glucose deprivation injury. Exp. Cell Res..

[27] Sharma A., Callahan L.M., Sul J.Y., Kim T.K., Barrett L., Kim M., Powers J.M., Federoff H., Eberwine J. (2010). A neurotoxic phosphoform of elk-1 associates with inclusions from multiple neurodegenerative diseases. PLoS ONE.

[28] Shi L., Kim A.J., Chang R.C., Chang J.Y., Ying W., Ko M.L., Zhou B., Ko G.Y. (2016). Deletion of mir-150 exacerbates retinal vascular overgrowth in high-fat-diet induced diabetic mice. PLoS ONE.

[29] Tan E., Ding X., Saadi A., Agarwal N., Naash M.I., Al-Ubaidi M.R. (2004). Expression of cone-photoreceptor-specific antigens in a cell line derived from retinal tumors in transgenic mice. Invest Ophthalmol. Vis. Sci..

[30] Livak K.J., Schmittgen T.D. (2001). Analysis of relative gene expression data using real-time quantitative pcr and the 2(-delta delta c(t)) method. Methods.

[31] Chang J.Y., Yu F., Shi L., Ko M.L., Ko G.Y. (2019). Melatonin affects mitochondrial fission/fusion dynamics in the diabetic retina. J. Diabetes Res..

[32] Shi L., Ko S., Ko M.L., Kim A.J., Ko G.Y. (2015). Peptide lv augments l-type voltage-gated calcium channels through vascular endothelial growth factor receptor 2 (vegfr2) signaling. Biochim. Biophys. Acta.

[33] Kim A.J., Chang J.Y., Shi L., Chang R.C., Ko M.L., Ko G.Y. (2017). The effects of metformin on obesity-induced dysfunctional retinas. Invest Ophthalmol. Vis. Sci..

[34] Rajagopal R., Bligard G.W., Zhang S., Yin L., Peter L., Clay F.S. (2016). Functional deficits precede structural lesions in mice with high-fat diet–induced diabetic retinopathy. Diabetes.

[35] Asare-Bediako B., Noothi S.K., Li Calzi S., Athmanathan B., Vieira C.P., Adu-Agyeiwaah Y., Dupont M., Jones B.A., Wang X.X., Chakraborty D. (2020). Characterizing the retinal phenotype in the high-fat diet and western diet mouse models of prediabetes. Cells.

[36] Bartel D.P. (2004). Micrornas: Genomics, biogenesis, mechanism, and function. Cell.

[37] Bartel D.P. (2009). Micrornas: Target recognition and regulatory functions. Cell.

[38] Wong-Riley M.T. (2010). Energy metabolism of the visual system. Eye Brain.

[39] Wan J., Yang J., Huang Y., Deng L. (2018). Microrna-150 inhibitors enhance cell apoptosis of melanoma by targeting pdcd4. Oncol. Lett..

[40] Ou H., Teng H., Qin Y., Luo X., Yang P., Zhang W., Chen W., Lv D., Tang H. (2020). Extracellular vesicles derived from microrna-150-5p-overexpressing mesenchymal stem cells protect rat hearts against ischemia/reperfusion. Aging.

[41] Ling Z., Fan G., Yao D., Zhao J., Zhou Y., Feng J., Zhou G., Chen Y. (2020). Microrna-150 functions as a tumor suppressor and sensitizes osteosarcoma to doxorubicin-induced apoptosis by targeting runx2. Exp. Ther. Med..

[42] Qin B., Shu Y., Xiao L., Lu T., Lin Y., Yang H., Lu Z. (2017). Microrna-150 targets elk1 and modulates the apoptosis induced by ox-ldl in endothelial cells. Mol. Cell. Biochem..

[43] Ying W., Tseng A., Chang R.C., Wang H., Lin Y.L., Kanameni S., Brehm T., Morin A., Jones B., Splawn T. (2016). Mir-150 regulates obesity-associated insulin resistance by controlling b cell functions. Sci. Rep..

[44] Demir O., Aysit N., Onder Z., Turkel N., Ozturk G., Sharrocks A.D., Kurnaz I.A. (2011). Ets-domain transcription factor elk-1 mediates neuronal survival: Smn as a potential target. Biochim. Biophys. Acta.

[45] Kawahara T., Shareef H.K., Aljarah A.K., Ide H., Li Y., Kashiwagi E., Netto G.J., Zheng Y., Miyamoto H. (2015). Elk1 is up-regulated by androgen in bladder cancer cells and promotes tumor progression. Oncotarget.

[46] Zhao H., Chen M., Wang J., Cao G., Chen W., Xu J. (2020). Pcna-associated factor kiaa0101 transcriptionally induced by elk1 controls cell proliferation and apoptosis in nasopharyngeal carcinoma: An integrated bioinformatics and experimental study. Aging.

[47] Halestrap A.P., McStay G.P., Clarke S.J. (2002). The permeability transition pore complex: Another view. Biochimie.

[48] Lavaur J., Bernard F., Trifilieff P., Pascoli V., Kappes V., Pages C., Vanhoutte P., Caboche J. (2007). A tat-def-elk-1 peptide regulates the cytonuclear trafficking of elk-1 and controls cytoskeleton dynamics. J. Neurosci..

[49] Wu W., Mosteller R.D., Broek D. (2004). Sphingosine kinase protects lipopolysaccharide-activated macrophages from apoptosis. Mol. Cell Biol..

[50] Wu D.M., Zhang Y.T., Lu J., Zheng Y.L. (2018). Effects of microrna-129 and its target gene c-fos on proliferation and apoptosis of hippocampal neurons in rats with epilepsy via the mapk signaling pathway. J. Cell Physiol..

[51] Fan L., Jiang L., Du Z. (2015). Myeloid cell leukemia 1 (mcl(-1)) protects against 1-methyl-4-phenylpyridinium ion (mpp+) induced apoptosis in parkinson’s disease. Metab. Brain Dis..

